# Non-typhoidal *Salmonella* myocarditis in an immunocompetent young adult with diarrhea

**DOI:** 10.11604/pamj.2019.34.117.19506

**Published:** 2019-10-29

**Authors:** Lamprini Markaki, Nikolaos Spernovasilis, Dimitris Lempidakis, Evangelos Kokorakis, Ioannis Gialamas, Stylianos Petousis, Diamantis Kofteridis, Emmanuel Simantirakis

**Affiliations:** 1 Department of Internal Medicine, University Hospital of Heraklion, Heraklion, Greece; 2 Department of Cardiology, University Hospital of Heraklion, Heraklion, Greece; 3 School of Medicine, University of Crete, Heraklion, Greece

**Keywords:** *Salmonella*, myocarditis, immunocompetent

## Abstract

Myocarditis is the inflammation of the heart muscle and it is caused by a wide range of infectious and non-infectious conditions. Non-typhoidal *Salmonella* infection, a common foodborne illness worldwide, only rarely causes myocarditis. We describe a case of an immunocompetent adult with *Salmonella enterica* serovar Typhimurium myocarditis who had a favorable outcome due to early recognition of the causative factor and prompt initiation of appropriate treatment.

## Introduction

According to the World Health Organization/International Society and Federation of Cardiology (WHO/ISFC), myocarditis is defined as an inflammatory disease of the myocardium and is diagnosed by established histological (Dallas criteria), immunological and immunohistochemical criteria [1]. This definition requires the performance of Endomyocardial Biopsy (EMB), which in clinical practice is infrequent, making the diagnosis of acute myocarditis challenging and its actual incidence difficult to determine [1]. An additional intriguing component of myocarditis management is the broad spectrum of its etiology, including infectious, immune-mediated and toxic causes, requiring individualized therapeutic approach, apart from the custom supporting measures [2]. Herein, we present an unusual case of acute non-typhoidal myocarditis caused by *Salmonella enterica* serovar Typhimurium (STM) in a previous healthy, immunocompetent adult male patient.

## Patient and observation

**Figure 1:**
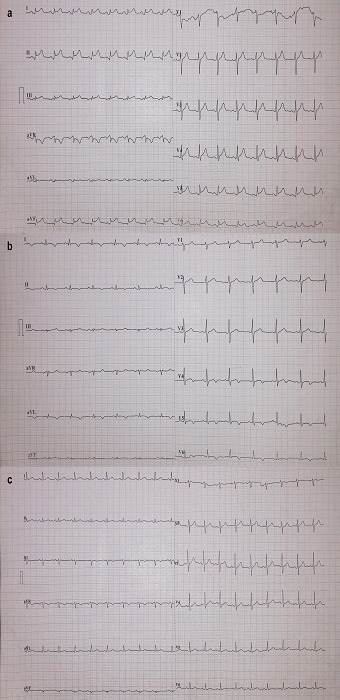
a) ECG on admission demonstrates mild respiratory sinus arrhythmia with a frequency of 95 beats per minute (bpm). Concave ST segment elevation in leads I, II, III, aVF, V4-V6 is obvious and no reciprocal ST segment depression of rest leads is detected, findings that could be associated with possible myocarditis (taking into account the elevated troponin and the echocardiogram findings). Note the presence of a non-typically ischemic q wave in inferior and lateral leads, potentially frustrating any treating doctor; b) ECG 4 days post-admission. Normal sinus rhythm 78 bpm. Note the progress of the initial repolarization disorders with all the previously affected leads now demonstrating T waves inversion. Minimal q waves can be noticed in I, aVL; c) Follow up ECG, two months after discharge. Normal sinus rhythm 85 bpm. Repolarization pattern is practically restored. T waves inverted in lead III and flattened in aVF are merely noted

A 39-year-old, previously healthy male, presented to the emergency department of our hospital with a 4-day history of watery, non-bloody diarrhea, fever up to 38.5°C and a 3-hour history of non-pleuritic, vague, retrosternal pain radiating to his left arm. He mentioned no regular medications and no recent travel. He and his child, who was suffering also from diarrhea but no fever, had ingested poultry prior to the onset of symptoms. On initial examination, he was febrile (38°C) while the rest of his vital signs were normal. Apart from increased bowel sounds, no abnormal physical findings were found. The chest x-ray was normal. Abnormal findings were noted on the Electrocardiogram (ECG) (Figure 1). Laboratory tests showed a moderate inflammatory syndrome and elevation of cardiac enzymes: troponin I 5,888 pg/ml (normal <19.8 pg/ml); Creatine Phosphokinase (CPK) 313 U/L; and Lactate Dehydrogenase (LDH) 305 U/L. Subsequently, the transthoracic Echocardiography (ECHO) revealed hypokinetic areas in the inferior and inferolateral segments of left ventricle and an Ejection Fraction (EF) of 40%. A coronary angiography was performed, without detectable coronary artery disease.

**Figure 2:**
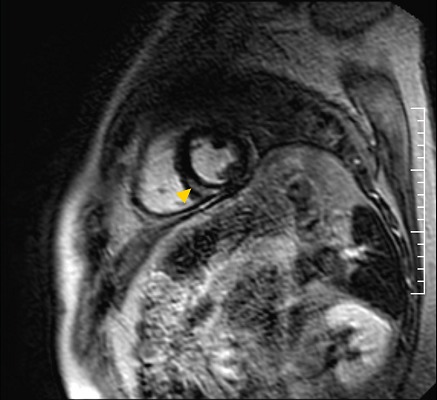
CMRI revealing an area with late gadolinium enhancement (arrowhead) at mid-wall myocardial layers of mid posteroseptal and inferior LV wall without accompanied pericardial enhancement

The patient was admitted in the cardiac intensive care unit with the diagnosis of clinically suspected acute myocarditis due to the elevated troponin and the initial findings on ECG and ECHO. On day 3 of hospitalization, STM was isolated from the stool culture and ciprofloxacin 400 mg i.v. bid was initiated. Blood cultures, obtained after the initiation of ciprofloxacin, were sterile. Tests for other infectious and non-infectious causes of myocarditis were negative. The patient improved within 72 hours of ciprofloxacin treatment, remaining asymptomatic during the rest of his hospitalization. There was a gradual fall of troponin, while ECG continued to be abnormal (Figure 1) but repeated ECHOs demonstrated normalization of left ventricular function. A Cardiac Magnetic Resonance Imaging (CMRI) on the day before patient discharge revealed findings which supported the diagnosis of acute myocarditis without pericardial involvement (Figure 2). The patient was discharged on day 12 with the diagnosis of acute myocarditis and instructions for an additional 5-day course of oral ciprofloxacin (500 mg bid) in order to complete 14 days of antibiotic treatment. At follow up, two months later, the patient was asymptomatic, in good health, and the ECG had returned to normal (Figure 1).

## Discussion

Myocarditis results from infectious and non-infectious causes, though often no cause can be identified [2, 3]. Viral infections predominate among other causes, whereas bacterial myocarditis is rare and usually seen in the context of severe sepsis or is associated with specific bacterial pathogens [4]. The non-typhoidal serovars of *Salmonella,* a gram-negative bacillus, are responsible only for a small number of cases in children and adults [5], while the exact pathogenesis remains obscure [6]. Nevertheless, the overall mortality in the reported cases is considerably high, around 20% [5]. The clinical presentation of acute myocarditis ranges from absence of symptoms to fatigue, chest pain, palpitations, dyspnea, life threatening arrhythmias, cardiogenic shock and sudden death [1, 3]. Remarkably, diarrhea has been reported in only around 20% of cases of non-typhoidal *Salmonella* myocarditis [5]. Although ECG changes are neither specific nor sensitive, its use is important in diagnosis and also prognosis of myocarditis. Though there are no pathognomonic findings, ECG analysis usually shows sinus tachycardia with non-specific ST segment changes and T wave abnormalities, while atrial, ventricular and intraventricular delays are common [3, 7]. Of note, only the presence of QRS prolongation has been associated with poorer prognosis [7].

Echocardiography remains a valuable tool in assessing the degree of cardiac dysfunction and in differential diagnosis. Findings include wall motion abnormalities and disturbances of ventricular function [8]. Our patient's initial ECHO revealed an EF <50% which is a risk factor for unfavorable outcome [8]. However, after administrating the proper antimicrobial therapy, his ECHO findings normalized, obviating the need for EMB and further supporting the causative association between *Salmonella* infection and myocarditis. CMRI is the pivotal non-invasive imaging technique for diagnosing myocarditis, monitoring disease progression and guiding EMB in certain cases, but often is not readily available and cannot characterize myocarditis histological types or be performed in hemodynamically unstable patients [1, 9]. The accuracy of standard CMRI techniques for the diagnosis of acute myocarditis is sufficient and it can be further improved with novel parametric mapping techniques [9]. The EMB remains the gold-standard method for diagnosis but sampling errors, inter-interpreter variability, complications associated with the procedure and low sensitivity, limit its use [3, 10]. In the context of our patient's clinical amelioration, EMB was thought to be excessive, underlying, once again, the necessity for non-invasive diagnostic methods that will be of low cost and easily accessible and performed.

## Conclusion

In conclusion, our case emphasizes the importance of considering non-typhoidal *Salmonella* as a cause of myocarditis, since the prompt initiation of appropriate antibiotic treatment is crucial in order to achieve a favorable outcome.

## Competing interests

The authors declare no competing interests.
